# Development and Validation of an Immune-Based Prognostic Risk Score for Patients With Resected Non-Small Cell Lung Cancer

**DOI:** 10.3389/fimmu.2022.835630

**Published:** 2022-03-23

**Authors:** Lan He, Yanqi Huang, Xin Chen, Xiaomei Huang, Huihui Wang, Yuan Zhang, Changhong Liang, Zhenhui Li, Lixu Yan, Zaiyi Liu

**Affiliations:** ^1^ Department of Radiology, Guangdong Provincial People’s Hospital, Guangdong Academy of Medical Sciences, Guangzhou, China; ^2^ Guangdong Provincial Key Laboratory of Artificial Intelligence in Medical Image Analysis and Application, Guangdong Provincial People’s Hospital, Guangdong Academy of Medical Sciences, Guangzhou, China; ^3^ The Second School of Clinical Medicine, Southern Medical University, Guangzhou, China; ^4^ Department of Radiology, Guangzhou First People’s Hospital, School of Medicine, South China University of Technology, Guangzhou, China; ^5^ Department of Radiology, the Third Affiliated Hospital of Kunming Medical University, Yunnan Cancer Hospital, Yunnan Cancer Center, Kunming, China; ^6^ Department of Pathology, Guangdong Provincial People’s Hospital, Guangdong Academy of Medical Sciences, Guangzhou, China

**Keywords:** non-small cell lung cancer, immune-based prognostic risk score, immunohistochemistry, overall survival, prognostic prediction

## Abstract

**Background:**

Despite the well-known role of immunoscore, as a prognostic tool, that appeared to be superior to tumor–node–metastasis (TNM) staging system, no prognostic scoring system based on immunohistochemistry (IHC) staining digital image analysis has been established in non-small cell lung cancer (NSCLC). Hence, we aimed to develop and validate an immune-based prognostic risk score (IMPRS) that could markedly improve individualized prediction of postsurgical survival in patients with resected NSCLC.

**Methods:**

In this retrospective study, complete resection of NSCLC (stage I–IIIA) was performed for two independent patient cohorts (discovery cohort, n=168; validation cohort, n=115). Initially, paraffin-embedded resected specimens were stained by immunohistochemistry (IHC) of three immune cell types (CD3+, CD4+, and CD8+ T cells), and a total of 5,580 IHC-immune features were extracted from IHC digital images for each patient by using fully automated pipeline. Then, an IHC-immune signature was constructed with selected features using the LASSO Cox analysis, and the association of signature with patients’ overall survival (OS) was analyzed by Kaplan–Meier method. Finally, IMPRS was established by incorporating IHC-immune signature and independent clinicopathological variables in multivariable Cox regression analysis. Furthermore, an external validation cohort was included to validate this prognostic risk score.

**Results:**

Eight key IHC-immune features were selected for the construction of IHC-immune signature, which showed significant associations with OS in all cohorts [discovery: hazard ratio (HR)=11.518, 95%CI, 5.444–24.368; validation: HR=2.664, 95%CI, 1.029–6.896]. Multivariate analyses revealed IHC-immune signature as an independent prognostic factor, and age, T stage, and N stage were also identified and entered into IMPRS (all *p*<0.001). IMPRS had good discrimination ability for predicting OS (C-index, 0.869; 95%CI, 0.861–0.877), confirmed using external validation cohort (0.731, 0.717–0.745). Interestingly, IMPRS had better prognostic value than clinicopathological-based model and TNM staging system termed as C-index (clinicopathological-based model: 0.674; TNM staging: 0.646, all *p*<0.05). More importantly, decision curve analysis showed that IMPRS had adequate performance for predicting OS in resected NSCLC patients.

**Conclusions:**

Our findings indicate that the IMPRS that we constructed can provide more accurate prognosis for individual prediction of OS for patients with resected NSCLC, which can help in guiding personalized therapy and improving outcomes for patients.

## Introduction

Non-small cell lung cancer (NSCLC) remains as the most common type of lung cancer, accounting for approximately 85% of all lung cancer patients (1). It is estimated that approximately 25% of NSCLC patients recur locally during follow-up despite optimal primary treatment (2). Recently, the major breakthroughs in immunotherapy and targeted therapy have brought substantially more effective treatment strategies for patients at high risk of recurrence (3, 4). Therefore, improved prognostication is still warranted to facilitate better postoperative management. The current tumor–node–metastasis (TNM) staging system provide the most reliable guidelines for routine prognostication and treatment of NSCLC (5). However, survival outcomes vary significantly among patients within the same TNM stage with similar treatment options (6). Therefore, there is evidently room for developing novel tools to improve the accuracy of survival prediction and facilitate personalized adjuvant treatment decision making to improve patient prognosis.

Additionally, it has been recognized that tumor-infiltrating lymphocytes (TIL) cells play important roles in tumor growth suppression depending on the density and location of various immune cell subpopulations for serial cancers including NSCLC (7). The heterogeneity of immune features may be the major reason for the difference in the prognosis of patients within the same TNM staging (8). Indeed, accumulating evidence suggests that quantification of intratumor immune infiltration in patients with NSCLC permits clinical interpretations regarding survival outcomes (6). Certain quantitative signature of tumor-infiltrating immune cells is increasingly recognized as a predictive biomarker to enable personalized treatment selection and improve patient management (9). Although the potential clinical relevance of density and ratios of infiltrating cells has already been evaluated in previous studies (6, 10–12), with the recent availability of high-throughput quantitative image features extracting algorithm (13), there is now an opportunity for the systematic analysis of immunohistochemistry (IHC) staining digital image to identify previously unrecognized features that correlate with patients’ prognoses (14). To the best of our knowledge, there were no prognostic models based on IHC-stained digital image analysis being developed to individually predict the survival outcomes in patients with resected NSCLC. In the present study, we aimed to improve the prognostic prediction of resected NSCLC through developing and validating an immune-based prognostic risk score (IMPRS) leveraging high-throughput features extracted from IHC-stained digital image.

## Materials and Methods

### Patients and Tissue Specimens

**Figure 1:**
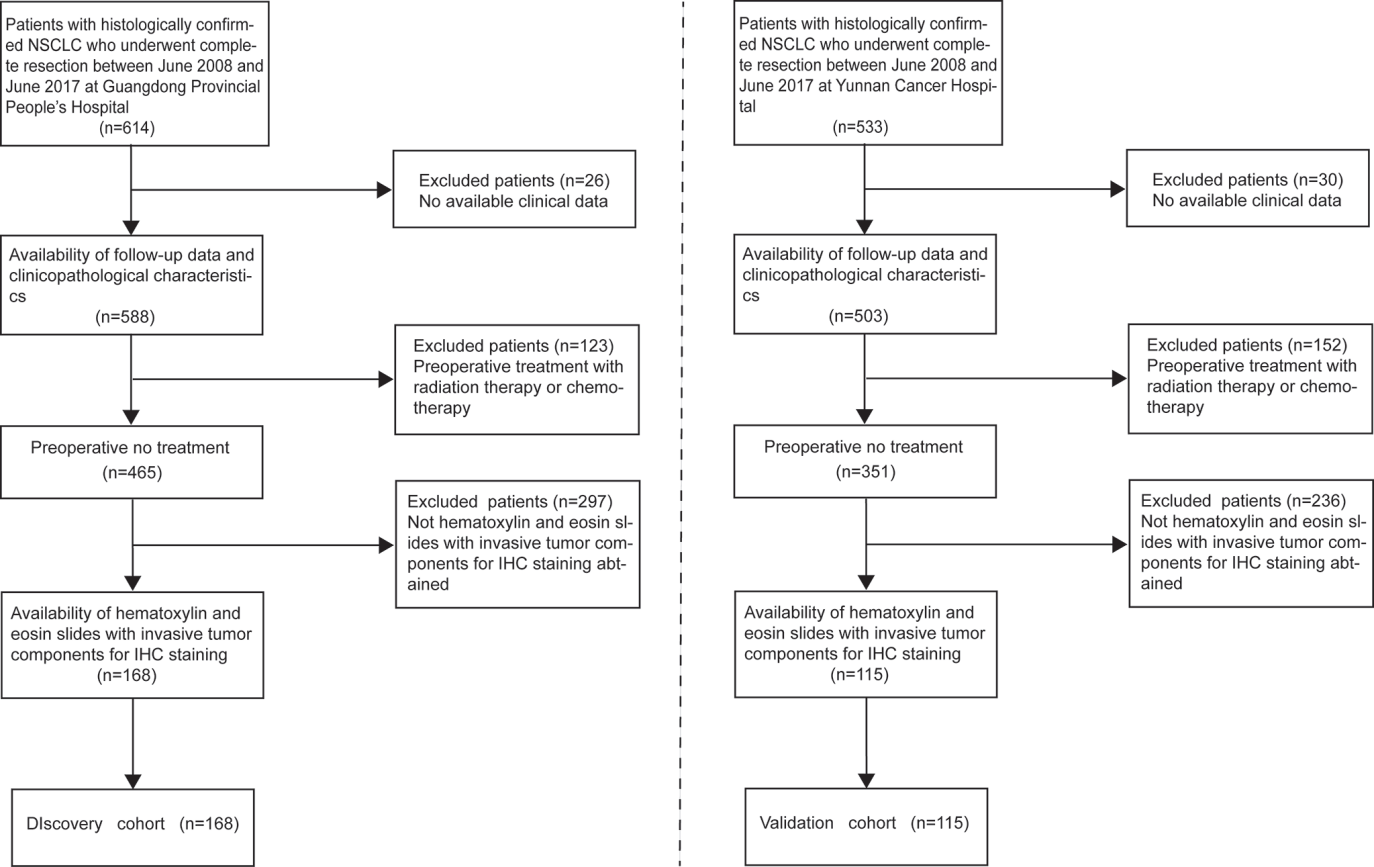
The flow diagram for patient recruitment process.

This retrospective study was approved by the Institutional Review Board of all participating institutions, and the requirement for informed consent was waived. We retrieved data of patients with primary biopsy-confirmed NSCLC who underwent complete resection between June 2008 and June 2017 at Guangdong Provincial People’s Hospital as the discovery cohort (n=168) for the development of IMPRS and patients at Yunnan Cancer Hospital between June 2008 and June 2017 as the validation cohort (n=115). The inclusion criteria were as follows: (1) patients with histopathological diagnosis of NSCLC; (2) patients with complete surgical resection of primary tumor; (3) patients with availability of hematoxylin and eosin slides with invasive tumor components for IHC staining; (4) patients with availability of follow-up data and clinicopathological characteristics; (5) patients with no history of cancer treatment; and (6) patients with no history of other types of cancer (**Figure 1**).

Clinicopathological variables, including age, sex, smoking status, histological type, and TNM stage, tumor grade, sampled LN number, surgical procedure, tumor location, and tumor diameter, were recorded for all eligible patients. The patients in the validation cohort were selected using the same criteria as those used for the discovery cohort. Primary endpoint of this study was overall survival (OS), which was defined as the time from surgery to death due to any cause (event) or last contact (censored). Our study was censored on July 1, 2017.

### Immunohistochemistry Staining

**Figure 2:**
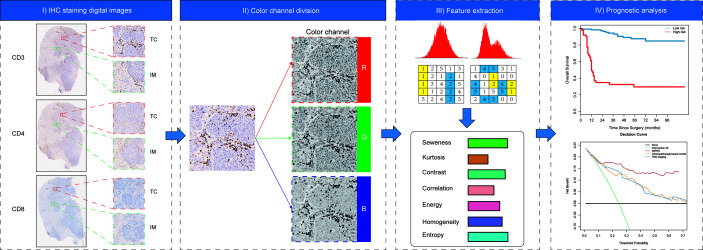
The workflow for the immunohistochemistry (IHC)-immune feature extraction.

On the basis of previous study findings (6), three prognostic immune biomarkers were selected for IHC staining: pan T cells (CD3) and cytotoxic T cells (CD4, CD8). Formalin-fixed paraffin embedded (FFPE) samples were constructed from tissue samples harvested on surgical specimens for IHC staining as described in **Appendix A1**, and three IHC digital images were attained for each patient. Then, the IHC digital images were evaluated by two independent pathologist who were blinded to the clinical outcome. The tissue sections were screened using an inverted research microscope (model DM IRB; Leica, Wetzlar, Germany) at a low power (×100), and then the most representative fields were selected. Thereafter, to extract the image features of IHC digital images by using fully automated pipeline, three respective areas of invasive margin (IM) and tumor center (TC) were measured at 200× magnification (**Figure 2**). Two pathologist, who were blinded to the clinicopathological characteristics, reviewed the stained slides to determine the IM and TC regions. Thereby, six types of IHC staining images for each patient were obtained including three types of T cells (CD3+, CD4+, and CD8+) and two regions (TC and IM). IHC staining was performed in the validation cohort using the same staining procedure, antibodies, and evaluation criteria as those used for the discovery cohort to allow for comparability.

### IHC-Immune Feature Extraction

All acquired IHC digital images were gathered for image feature extraction using fully automated pipeline (**Figure 2**). Each color IHC staining image was split into three independent RGB channel images and thereafter were used for further feature extraction. The details of the image features extraction algorithms are summarized in **Appendix A2**. To ensure the reproducibility and accuracy, we performed a reproducibility analysis using the inter- and intraclass correlation coefficients (ICCs) for IHC-immune feature extraction. Two pathologists with 15 years (observer 1) and 12 years (observer 2) of experience in lung cancer performed image acquisition and selection of fields of view for IHC-immune feature extraction procedure, in a blind fashion. Then, observer 1 repeated the procedure with an interval of 1 week for the assessment of the intraobserver agreement of IHC-immune feature extraction. A ICC >0.75 indicated a good agreement. In total, 5,580 quantitative features were extracted for each patient, which included intensity and texture features (**Appendix Table A1**).

### Feature Selection and Construction of IHC-Immune Signature

First, data of IHC-immune features were normalized with Z-score normalization. Then, the least absolute shrinkage and selection operator method (LASSO)-penalized Cox regression was exploited to select the most important IHC-immune features for the IHC-immune signature construction using the discovery cohort. Subsequently, IHC-immune signature was developed by integrating selected features with the corresponding regression coefficients from the LASSO Cox regression analysis. Time-dependent receiver operating characteristic (ROC) curves was applied to obtain the optimal cutoff values of IHC-immune signature for providing the best separation between the groups of patients (high risk vs. low risk) related to their OS outcome, and the generated group was analyzed by the Kaplan–Meier method with log-rank test.

### Construction and Validation of IMPRS

The prognostic value of IHC-immune signature for predicting OS was assessed in both discovery and validation cohorts using concordance index (C-index). We then combined IHC-immune signature with all available clinical and pathological variables in the multivariate Cox regression analyses for model building. The following 12 potential prognostic factors were examined in univariate analysis for their possible association with OS: T stage, N stage, age, histological type, gender, smoking status, sampled LN number, surgical procedure, tumor location, tumor diameter, tumor grade, and IHC-immune signature. A statistical relationship with OS (*p*<0.3) was used as a criterion to retain factors that could have a potential significant impact on OS predicting. Then, a final model was selected using a backward step-down process, which used the Akaike information criterion as a stopping rule. Based on the results of the final model, a prognostic scoring system named IMPRS was formulated for individualized probability prediction of OS. The performance of IMPRS was measured by the C-index, the integrated area under the ROC curve (iAUC), and the integrated Brier score (iBS), and also was assessed by comparing the predicted versus observed survival probability using the calibration curve. In addition, external validation was further performed to assess the prognostic predictive power of IMPRS with an independent validation cohort.

### Clinical Usefulness of IMPRS

To evaluate the clinical benefits of this established IHC-immune signature, another clinicopathological-based model was also constructed with only clinicopathological factors, and TNM staging system was constructed by combining T and N stages. Then, we compared the prognostic performance for predicting OS between IMPRS and clinicopathological-based model or TNM staging system by using C-index, iAUC, iBS, net reclassification improvement (NRI), and integrated discrimination improvement (IDI). The larger C-index with positive value for NRI and IDI indicated the more accurate performance for predicting OS. Furthermore, the higher value of iAUC and the lower value of iBS indicated the better performance for predicting OS. Finally, a decision curve analysis was performed to determine the clinical usefulness of IMPRS by quantifying the net benefits at different threshold probabilities.

Furthermore, the stratified analyses were performed to investigate the potential association of the IHC-immune signature with OS using subgroups within stage I NSCLC patients and patients that received adjuvant chemotherapy and those that did not receive adjuvant chemotherapy. The performances of the constructed IMPRS for OS prediction were, meanwhile, investigated for those subgroups.

### Statistical Analysis

This study adhered to the transparent reporting of a multivariable prediction model for individual prognosis or diagnosis (TRIPOD) statement for reporting (15). Statistical analysis was performed using the R software (version 3.2.4, http://www.Rproject.org), and the details of all R packages used in this study are described in **Appendix A3**. The survival outcomes were analyzed by the Kaplan–Meier curves, and the log-rank test was used to compare differences in survival curves. All tests were two-sided, and a *p* < 0.05 was considered statistically significant.

## Results

### Clinicopathological Characteristics of Patients

A total of 283 patients with resected NSCLC (discovery cohort: n=168; external validation cohort: n=115) were included in the analysis, and the details of patients’ clinicopathological characteristics are shown in **Table 1**. In brief, the discovery cohort had 64 female and 104 male patients, while in the validation cohort, there were 58 male and 57 female patients. Forty-five patients had received adjuvant chemotherapy in the discovery cohort, while 62 patients had received adjuvant chemotherapy in the validation cohort. Within the study population, the median follow-up times were 53.0 [interquartile range (IQR), 16.7–72.7] months in the discovery cohort and 58.0 (IQR, 36.0–67.0) months in the validation cohort, respectively.

**Table 1:** Characteristics of patients in the discovery cohort and validation cohort.

Characteristic	Discovery cohort (GDPH; n = 168)	Validation cohort (YCH; n = 115)
**Age [y, Median (IQR)]**	61.0 (55.3, 67.8)	55.0 (48.0, 64.0)
**Sex**		
Male	104 (61.9%)	58 (50.4%)
Female	64 (38.1%)	57 (49.6%)
**Smoking Status**		
Yes	53 (31.5%)	38 (33.0%)
No	115 (68.5%)	77 (67.0%)
**Histological type**		
Adenocarcinoma	123 (73.2%)	104 (90.4%)
Others	45 (26.8%)	11 (9.6%)
**T stage**		
T1	60 (35.7%)	82 (71.3%)
T2	80 (47.6%)	24 (20.9%)
T3	26 (15.5%)	6 (5.2%)
T4	2 (1.2%)	3 (2.6%)
**N stage**		
N0	122 (72.6%)	85 (73.9%)
N1	16 (9.5%)	12 (10.4%)
N2	30 (17.9%)	18 (15.7%)
**TNM stage**		
IA+IB	105 (62.5%)	74 (64.3%)
IIA+IIB	27 (16.1%)	21 (17.2%)
IIIA	36 (21.4%)	20 (17.5%)
**Sampled LN number [n, Median (IQR)]**	18 (13, 25)	12 (7, 16)
**Surgical procedure**		
Pneumonectomy	19 (11.3%)	5 (4.3%)
Lobectomy/bilobectomy	118 (70.2%)	79 (68.7%)
Segmentomy	22 (13.1%)	24 (20.9%)
Wedge	9 (5.4%)	7 (6.1%)
**Tumor location**		
Right upper lobe	64 (38.1%)	31 (27.0%)
Right middle lobe	13 (7.7%)	6 (5.2%)
Right lower lobe	29 (17.3%)	32 (27.8%)
Left upper lobe	39 (23.2%)	27 (23.5%)
Left lower lobe	23 (13.7%)	19 (16.5%)
**Tumor diameter [cm, Median (IQR)]**	3.0 (1.8, 4.0)	2.2 (1.5, 4.0)
**Tumor grade**		
Well differentiated	9 (5.4%)	3 (2.6%)
Moderately differentiated	114 (67.9%)	80 (69.6%)
Poorly differentiated	45 (26.8%)	32 (27.8%)
**Received adjuvant chemotherapy**		
Yes	45 (26.8%)	62 (53.9%)
No	109 (64.9%)	53 (46.1%)
Unknown	14 (8.3%)	NA
**Follow-up time**		
Median	53.0	58.0
IQR	(16.7, 72.7)	(36.0, 67.0)

y, years; n, numbers; IQR, interquartile range; NA, not available; GDPH, Guangdong Provincial People’s Hospital; YCH, Yunnan Cancer Hospital.

### Impact of IHC-Immune Signature

**Figure 3:**
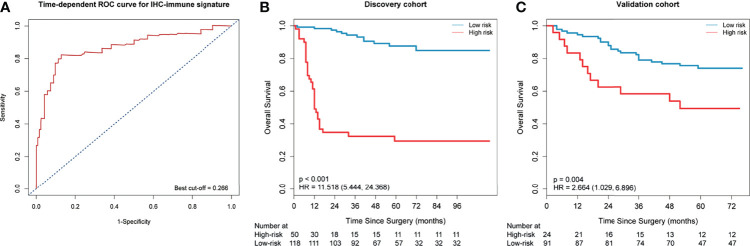
Time-dependent receiver-operating characteristic (ROC) curve and Kaplan–Meier survival curves. **(A)** Time-dependent ROC curve at 3 years in the discovery cohort. Patients were stratified into high- or low-risk group based on the cutoff value (cutoff=0.266). Kaplan–Meier survival curves for overall survival (OS) in the discovery **(B)** and validation cohort **(C)** according to the immunohistochemistry (IHC)-immune signature.

In the reproducibility analysis of IHC-immune feature extraction, the interobserver ICCs were satisfactory, which ranged from 0.750 to 0.980 based on observer 1’s and observer 2’s first IHC-immune feature extraction, and the intraobserver ICCs also remained satisfactory, which ranged from 0.758 to 0.921 based on observer 1’s twice IHC-immune feature extractions. Thus, a total of 5,580 IHC-immune features were finally extracted from all IHC-based digital images for each patient. After the normalization of features, eight key features with non-zero coefficients were selected in the LASSO Cox regression analysis (**Appendix Figure A1**), and then, the IHC-immune signature was constructed with a corresponding value calculated for each patient (**Appendix A4**). The cutoff value of the IHC-immune signature was 0.266, which was generated by a time-dependent ROC curve (**Figure 3A**). Thus, 118 patients were classified into low-risk group, and 50 patients were assigned to high-risk group in the discovery cohort (**Figure 3B**). Ninety-one patients were classified into low-risk group, and 24 patients were assigned to high-risk group in the validation cohort (**Figure 3C**). Subsequently, patients in the low-risk group exhibited higher OS rates than those in the high-risk group in discovery (HR=11.518, 95%CI, 5.444–24.368) and validation cohort (HR=2.664; 95%CI, 1.029–6.896). In the discovery cohort, 3-year OS rates of patients were 0.943 (95%CI, 0.900–0.989) in the low-risk group and 0.324 (95%CI, 0.216–0.487) in the high-risk group (p<0.001), while in the validation cohort, 3-year OS rates of patients were 0.790 (95%CI, 0.710–0.878) in the low-risk group and 0.583 (95%CI, 0.416–0.818) in the high-risk group (p=0.004). Different prognostic strata in OS with a high statistical significance was showed in Kaplan–Meier curves between the high- and low-risk survival subgroups in all cohorts (all *p*<0.01, **Figures 3B, C**). Furthermore, the C-index of IHC-immune signature for predicting OS was 0.824 (95%CI, 0.815–0.833) in the discovery cohort and 0.708 (95%CI, 0.694–0.722) in the validation cohort.

### IMPRS for Predicting of OS

**Figure 4:**
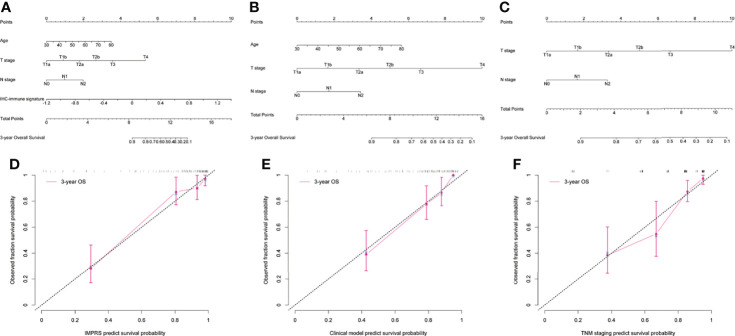
Visualization and calibration of the prognostic model. **(A–C)** The visualization of the prognostic model as a nomogram for patients with resected NSCLC (**A** for IMPRS, **B** for clinicopathologic-based model, **C** for TNM staging system). **(D–F)** The calibration curves for predicting overall survival (OS) at each time point (**D** for IMPRS, **E** for clinicopathologic-based model, **F** for TNM staging system).

In the univariate analysis, 10 potential prognostic factors showed significant association with OS (p<0.3), which were selected to be included in the multivariable analysis (**Table 2**). When combining IHC-immune signature with the selected potential prognostic factors, only IHC-immune signature, age, T stage, and N stage remained significant for predicting OS in the multivariable analysis (**Table 3**). A strong impact of IHC-immune signature on the prediction of OS was also evidenced (HR, 9.121; 95%CI, 4.354–19.108, p<0.001). Based on the results of multivariable analysis, an individual prognostic scoring system named IMPRS, which incorporated the significant prognostic factors, was established (**Figure 4A**; **Table 3**). For comparison of IMPRS to predict OS, clinicopathological-based model with only clinicopathological factors (with age, T stage, and N stage) and TNM staging system (with T stage and N stage) was also constructed (**Figures 4B, C**; **Table 3**).

**Table 2:** Univariate analyses for the potential prognostic predictors.

Variable	Coefficient	HR (95%CI)	*p*
**T stage**	1.336	3.805 (2.497, 5.798)	<0.001*
**N stage**	0.834	2.303 (1.685, 3.146)	<0.001*
**Age**	0.031	1.031 (0.997, 1.067)	0.071*
**Histological type**	-0.661	0.517 (0.241, 1.108)	0.090*
**Gender**	-0.452	0.636 (0.339, 1.193)	0.159^*^
**Smoking status**	0.538	1.713 (0.952, 3.082)	0.073^*^
**Sampled LN number**	0.012	1.012 (0.985, 1.041)	0.388
**Surgical procedure**	-0.345	0.709 (0.442, 1.135)	0.152^*^
**Tumor location**	-0.093	0.911 (0.753, 1.102)	0.336
**Tumor diameter**	0.115	1.122 (1.043, 1.206)	0.002^*^
**Tumor grade**	0.784	2.190 (1.264, 3.792)	0.005^*^
**IHC-immune signature**	2.908	18.313 (8.622, 38.894)	<0.001*

HR, hazard ratio; IHC, immunohistochemistry; CI, confidence interval.

*A statistical relationship with OS (p < 0.30) was used as a criterion to retain factors that could have a potential significant impact on OS prediction.

**Table 3:** Multivariate Cox proportional hazard analysis for prediction of OS among patients with resected NSCLC.

Variable	Unadjusted stratified Cox model			IMPRS			clinicopathologic-based model			TNM staging system		
	Coefficient	HR (95%CI)	*p*	Coefficient	HR (95%CI)	*p*	Coefficient	HR (95%CI)	*p*	Coefficient	HR (95%CI)	*p*
**T stage**	0.960	2.611 (1.469, 4.641)	0.001	1.023	2.781 (1.728, 4.478)	<0.001	1.180	3.254 (2.044, 5.180)	<0.001	1.181	3.259 (2.061, 5.153)	<0.001
**N stage**	0.580	1.787 (1.260, 2.535)	0.001	0.567	1.763 (1.254, 2.477)	0.001	0.606	1.832 (1.324, 2.535)	0.0003	0.580	1.787 (1.292, 2.472)	0.0005
**Age**	0.054	1.056 (1.016, 1.097)	0.005	0.040	1.041 (1.008, 1.075)	0.015	0.040	1.041 (1.006, 1.077)	0.023	—	—	—
**Histological type**	-0.857	0.452 (0.184, 0.978)	0.044	—	—	—	—	—	—	—	—	—
**Gender**	0.246	1.279 (0.586, 2.792)	0.537	—	—	—	—	—	—	—	—	—
**Smoking status**	-0.581	0.559 (0.251, 1.246)	0.155	—	—	—	—	—	—	—	—	—
**Surgical procedure**	-0.055	0.947 (0.594, 1.510)	0.818	—	—	—	—	—	—	—	—	—
**Tumor diameter**	0.092	1.096 (0.940, 1.277)	0.241	—	—	—	—	—	—	—	—	—
**Tumor grade**	0.543	1.721 (0.950, 3.116)	0.073	—	—	—	—	—	—	—	—	—
**IHC-immune signature**	2.366	10.653 (4.723, 24.028)	<0.001	2.211	9.121 (4.354, 19.108)	<0.001	—	—	—	—	—	—

IMPRS, immune-based prognostic risk score; HR, hazard ratio; IHC, immunohistochemistry; CI, confidence interval.

The score value of IMPRS was calculated as follow: Score = 1.023 × T stage + 0.567 × N stage + 0.040 × Age + 2.211 × IHC-immune signature. The score value of clnicopathological-based model was calculated as follow: Score = 1.180 × T stage + 0.606 × N stage + 0.040 × Age. The score value of TNM staging system was calculated as follows: Score = 1.181 × T stage + 0.580 × N stage.

**Figure 5:**
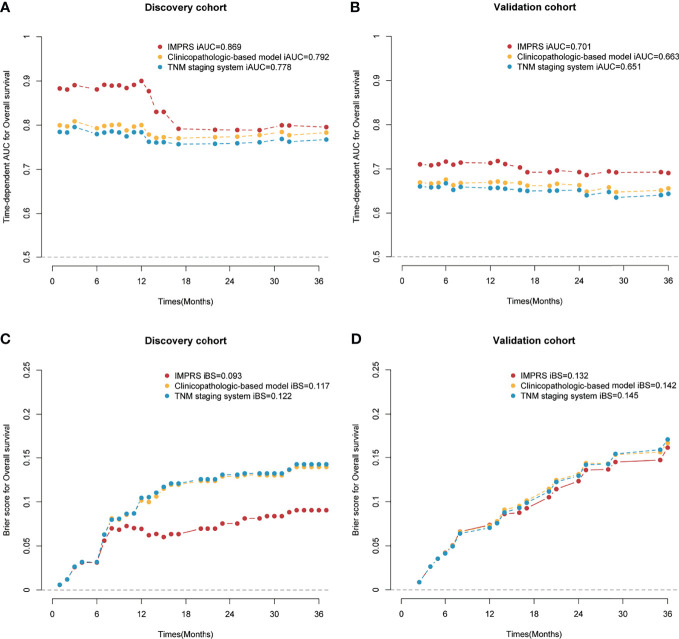
Time-dependent AUC and Brier score for overall survival (OS) in the discovery and validation cohort. **(A)** Time-dependent AUC for the discovery cohort; **(B)** time-dependent AUC for the validation cohort; **(C)** Brier score for the discovery cohort; **(D)** Brier score for the validation cohort.

**Figure 6:**
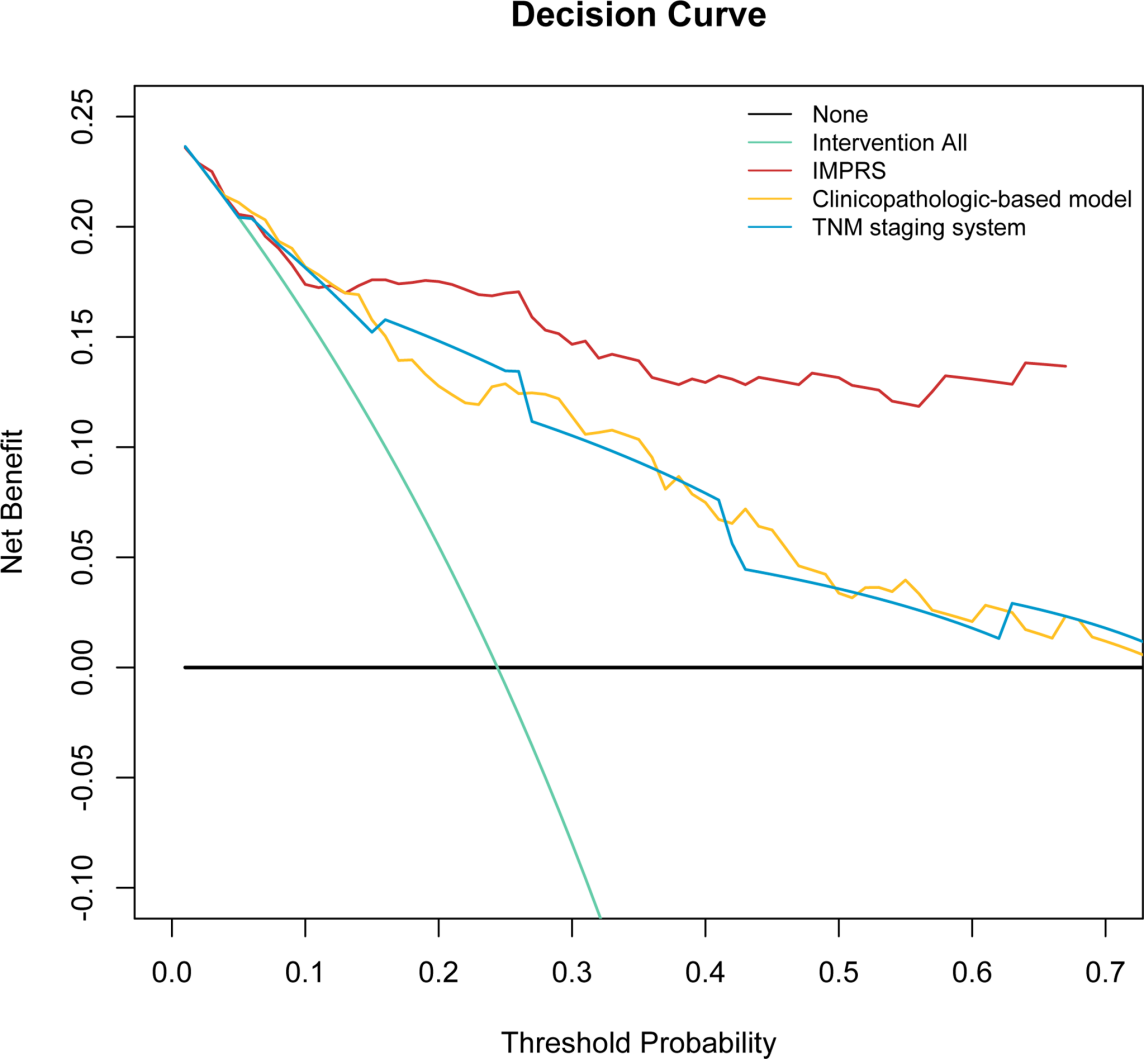
Decision curve analysis for the comparison of the IMPRS (red line), clinicopathological-based model (orange line), and TNM staging system (blue line) in term of clinical usefulness. The y-axis measures the net benefit.

In the discovery cohort, the Harrell’s C-index for the established IMPRS to predict OS (0.869; 95%CI, 0.861–0.877) was significantly higher than that of the clinicopathological-based model (0.814; 95%CI, 0.805–0.823) and TNM staging system (0.786; 95%CI, 0.776–0.796) (*p*<0.05, **Table 4**). In the validation cohort, the C-index of IMPRS (0.731; 95%CI, 0.717–0.745) was also greater than that of the clinicopathological-based model (0.674; 95%CI, 0.657–0.691) and TNM staging system (0.646; 95%CI, 0.626–0.666) (*p*<0.05, **Table 4**) for OS prediction. The calibration plots presented good agreement between the model prediction and actual observation for OS (all *p*>0.05; **Figures 4D–F**). The iAUC of IMPRS were significantly higher than that of clinicopathological-based model and TNM staging system in both discovery (IMPRS, 0.869; clinicopathological-based model, 0.792; TNM staging system, 0.778) and validation cohorts (IMPRS, 0.701; clinicopathological-based model, 0.663; TNM staging system, 0.651) (**Figures 5A, B**; all *p*<0.05). The iBS of IMPRS were significantly lower than that of clinicopathological-based model in both discovery (IMPRS, 0.092; clinicopathological-based model, 0.117; TNM staging system, 0.122) and validation cohort (IMPRS, 0.132; clinicopathological-based model, 0.142; TNM staging system, 0.145) (**Figures 5C, D**; all *p*<0.05). Consequently, IMPRS showed as a more accurate and useful tool based on IHC staining digital image analysis compared with the clinicopathological-based model for the prediction of OS regarding the NRI of 0.534 (95%CI, 0.179–0.684) in discovery and 0.418 (95%CI, 0.058–0.591) in validation cohorts, and IDI of 0.229 (95%CI, 0.090–0.363) in discovery and 0.050 (95%CI, 0.003–0.109) in validation cohorts (all *p*<0.05). In addition, IMPRS showed as a more accurate and useful tool compared with TNM staging system for the prediction of OS regarding the NRI of 0.790 (95%CI, 0.618–0.894) in discovery and 0.267 (95%CI, 0.015–0.526) in validation cohorts, and IDI of 0.022 (95%CI, 0.004–0.205) in discovery and 0.058 (95%CI, 0.008–0.142) in validation cohorts (all *p*<0.05). Furthermore, decision curve analysis showed that IMPRS had a higher overall net benefit than clinicopathological-based model and TNM staging system across a range of risk thresholds (**Figure 6**).

**Table 4:** Prediction performance of IHC-immune signature, IMPRS, and clinicopathological-based model in all patient cohort.

	Discovery cohort				Validation cohort			
	C-index (95%CI)	AUC at 3 years (95%CI)	iAUC	iBS	C-index (95%CI)	AUC at 3 years (95%CI)	iAUC	iBS
**IHC-immune signature**	0.824 (0.815–0.833)	0.858 (0.779–0.938)	0.818	0.138	0.708 (0.694–0.722)	0.774 (0.688–0.861)	0.617	0.150
**TNM staging system**	0.786 (0.776–0.796)	0.810 (0.731–0.888)	0.778	0.122	0.646 (0.626–0.666)	0.653 (0.523–0.783)	0.651	0.145
**Clinicopathologic-based model**	0.814 (0.805–0.823)	0.827 (0.754–0.900)	0.792	0.117	0.674 (0.657–0.691)	0.694 (0.576–0.812)	0.663	0.142
**IMPRS**	0.869 (0.861–0.877)	0.893 (0.826–0.961)	0.869	0.093	0.731 (0.717–0.745)	0.785 (0.694–0.877)	0.701	0.132

IMPRS, immune-based prognostic risk score; IHC, immunohistochemistry; CI, confidence interval; iAUC, the integrated area under the ROC curve; iBS, the integrated Brier score.

### Stratified Analysis of IMPRS in Patients With Stage I NSCLC and Patients Received Adjuvant Chemotherapy

To further investigate the clinical benefits of this IMPRS, two subgroups of patients with stage I NSCLC and patients who received adjuvant chemotherapy were used to validate the performance for OS prediction. A total of 105 patients had stage I NSCLC in the discovery cohort, while 74 patients had stage I NSCLC in the validation cohort. Consequently, IHC-immune signature showed significant associations with OS in patients with stage I NSCLC (discovery: HR=4.294; 95%CI, 1.208–15.260, *p*=0.024; validation: HR=3.012; 95%CI, 1.005–9.027, *p*=0.050) (**Appendix Figure A2**). IMPRS also showed good discrimination ability for predicting OS in patients with stage I NSCLC (discovery: C-index=0.732; 95%CI 0.701–0.763; validation: C-index=0.663; 95%CI, 0.636–0.690).

Furthermore, IHC-immune signature also remained to have significant associations with OS in patients who received adjuvant chemotherapy (discovery: HR=8.628; 95%CI, 2.520–29.538, *p*<0.001; validation: HR=2.441; 95%CI, 1.001–9.283, *p*=0.050) and patients who did not (discovery: HR=12.560; 95%CI, 4.446–35.480, *p*<0.001; validation: HR=3.453; 95%CI, 1.109–10.751, *p*=0.033) (**Appendix Figure A3**). IMPRS also showed good discrimination ability for predicting OS in patients that received adjuvant chemotherapy (discovery: C-index=0.779; 95%CI, 0.616–0.942; validation: C-index=0.707; 95%CI 0.683–0.731) and patients who did not receive adjuvant chemotherapy (discovery: C-index=0.906; 95%CI, 0.893–0.919; validation: C-index=0.778; 95%CI, 0.742–0.814).

## Discussion

In this study, we designed an automated pipeline that extracted quantitative image features from the IHC digital images, then built and evaluated an IHC-immune signature to distinguish NSCLCs with different survival outcomes, and ultimately developed and validated an immune-based prognostic scoring system, designated as IMPRS, to predict the individualized survival outcomes of NSCLC patients. To our knowledge, this is the first study to show the utility of high throughput image features extracted from immune-related IHC digital images to predict patient survival in resected NSCLC patients. Decision curve analysis revealed that IMPRS had better prognostic value than clinicopathological-based model and TNM staging system. As such, it could provide individualized survival prediction for improved patient prognosis. Furthermore, we also had validated the performance of the proposed IMPRS for OS prediction in patients with stage I NSCLC, and patients who received adjuvant chemotherapy and those who did not. The results showed that IHC-immune signature also remained to have significant associations with OS in such subgroups, which revealed that the IMPRS could potentially identify a subgroup of patients with worse prognosis that could require more close surveillance or even propose for adjuvant chemotherapy.

There is serial evidence that the tumor microenvironment of NSCLC is rich in different types of immune cells, which are associated with clinical outcomes (6, 11, 16). For example, the immunoscore was proposed based on the density of stromal CD8+ TILs and has been validated as a risk assessment tool in resected NSCLC, highlighting the potential importance of evaluating the immune infiltrate of tumor in guiding decision-making in the clinic (10). Kayser et al. had explored that the subset of stromal CD4+/CD25+ T cells was an independent prognostic marker in NSCLC patients (17). Paulsen et al. proposed CD45RO as a candidate marker for TNM immunoscore in the squamous cell carcinoma NSCLC (12). Boscolo et al. constructed the combined immunoscore (CD8+, CD4+, and CD68) for prognostic stratification of early stage NSCLC (18). However, these methods were limited to the density or the ratio of immune cells for the construction of immunoscore, which could not comprehensively explore the potential information for the heterogeneity of immune features in resected NSCLC. Tumor-immune interaction remains to be a potential prognostic factor in NSCLC (19). In this study, we presented a digital IHC images-based signature to predict OS for patients with resected NSCLC. We showed that the established IHC-immune signature constructed by quantitative features extracted from digital IHC images, which can potentially capture biologic properties of immune heterogeneities, can provide prognostic information for OS prediction. We can successfully stratify patients into high- and low-risk recurrence group and demonstrate that approximately 20%–30% of patients were predicted as high-risk recurrence using the IHC-immune signature. Patients in low-risk recurrence group exhibited higher OS rates than patients in high-risk recurrence group (all *p*<0.05).

The IHC-immune signature that we proposed has advantages over the previous methods, which were based on the immune-related genes. Li et al. developed an immune signature that can estimate prognosis in patients with early-stage non-squamous NSCLC by using immune-related genes and achieved a C-index of 0.64 (9). Öjlert et al. had only investigated if the tumor immune microenvironment showed association with prognosis after surgery in lung adenocarcinoma or lung SCC by using NSCLC gene expression and PD-L1 expression (20). Mi et al. developed the prognostic immune signatures based on immune-related genes by using The Cancer Genome Atlas (TCGA) samples, which can stratify patients into high- and low-risk group (21). Sun et al. developed TIL-associated IncRNA signature for predicting 3-year OS and yielded an AUC of 0.646 (22), and Zhuang et al. developed immune gene risk index for predicting 3-year OS with an AUC of 0.666 (23). However, no individual prognostic models in those study were developed to predict the individualized survival outcomes of NSCLC patients. Through our constructed IMPRS, clinicians could more precisely estimate the survival of individual patients after surgery and identify subgroups of patients who were in need of a specific treatment strategy. We also presented the IMPRS as a nomogram, which could be used as an easy-to-use tool to attain individualized probability scoring of OS in patients with resected NSCLC. Tian et al. obtained immune gene prognostic models for lung adenocarcinoma (LUAD) and lung squamous cell carcinoma (LUSC) based TCGA database by using gene expression and achieved the AUCs of 0.742, 0.707, and 0.711 for LUAD, and 0.668, 0.703, and 0.668 for LUSC (24). In our study, the proposed IMPRS integrated IHC-immune signature based on immune-related IHC digital images and clinicopathological factors (age, T stage, and N stage) yielded a C-index of 0.869, demonstrating a better prediction performance than that achieved by their studies.

The multivariate analyses revealed that IHC-immune signature remained as an independent indicator for prognosis in all cohorts. However, besides IHC-immune signature, age, gender, smoking history, histological type, T stage, and N stage, tumor grade, sampled LN number, surgical procedure, tumor location, and tumor diameter might also have important prognostic significance for OS predicting. Thus, we combined IHC-immune signature and these clinicopathological factors to better predict the prognosis of resected NSCLC patients. In the results, we identified that age, T stage, and N stage remained as independent prognostic factors. These findings were in high concordance with previous studies for resected NSCLC patients (25). Gao et al. had developed an immunoscore-based prognostic nomogram-integrated macrophage immunoscore (CD68 and CD163), lymphocyte-to-monocyte ratio, and TNM stage for OS prediction termed as a C-index of 0.810 (26). In our study, the proposed IMPRS-integrated IHC-immune signature and clinicopathological factors (age, T stage, and N stage) yielded a C-index of 0.869, demonstrating a better prediction performance than that achieved by Gao et al.

To further investigate how much extra clinical benefits we can obtain for individualized OS prediction by incorporating IHC-immune signature, we also developed and compared the clinicopathological-based model (with age, T stage, and N stage) and TNM staging system (with T stage and N stage). Finally, the results showed that the IMPRS showed better discrimination performance than the clinicopathological-based model and TNM staging system in all cohorts (*p*<0.05, **Table 4**). The decision curve analysis proved that IMPRS offered significant improvement for individualized OS prediction comparing with clinicopathological-based model and TNM staging system.

Although the proposed IMPRS demonstrated good levels of accuracy for predicting OS, there are some limitations to our study. That is, the limited immune biomarkers that we used were restricted to CD3+, CD4+, and CD8+ IHC digital images features, and other potential immune biomarkers, such as CD45RO, CD20, and PDL-1, were not included as variables in the model analysis. However, the traditional IHC immunoscoring approach remains suboptimal because of the lack of a consistent standard (6, 11). With regard to other immune cells, particularly CD45RO cells, recent studies revealed that its prognostic values remained uncertain due to background staining and loss of antigenicity in stored sections (6). Thus, we selected the IHC staining of CD3, CD4, and CD8 cells between TC and IM regions for immune infiltration analysis. Our established IHC-immune signature utilizes eight IHC-immune features, which were significantly associated with OS for resected NSCLC. Determined by CD8+ cells in the TC and IM and CD4+ cells in the TC, the immunoscore was an excellent prognostic factor for resected NSCLC, which was consisted with serial studies (6, 10). Through this constructed IMPRS, clinicians could more precisely estimate the survival of individual patients after surgery and identify subgroups of patients who were in need of a specific treatment strategy. We presented the IMPRS as a nomogram, which could be used as an easy-to-use tool to attain individualized probability scoring of OS in patients with resected NSCLC. However, another limitation of our study was that all procedures were performed on a laptop. To make this more usable in clinical practice, it is an interesting attempt to make this algorithm as an App in the future for Smartphones that could be more convenient to clinicians.

In conclusion, we established and validated a novel IMPRS incorporating the IHC-immune signature and clinicopathological factors (age, T stage, and N stage) for predicting survival of patients with resected NSCLC. Through this model, clinicians could more precisely estimate the survival of individual patients after surgery and identify subgroups of patients who were in need of a specific treatment strategy.

## Data Availability Statement

The raw data supporting the conclusions of this article will be made available by the authors, without undue reservation.

## Ethics Statement

The studies involving human participants were reviewed and approved by Research Ethics Committee of Guangdong Provincial People’s Hospital, Guangdong Academy of Medical Sciences. Written informed consent to participate in this study was provided by the participants’ legal guardian/next of kin. Written informed consent was obtained from the individual(s) and minor(s)’ legal guardian/next of kin for the publication of any potentially identifiable images or data included in this article.

## Author Contributions

Conception and design: ZYL and LH. Administrative support: ZYL. Provision of study materials or patients: ZYL, LXY, and ZHL. Collection and assembly of data: LH, YQH, and XC. Data analysis and interpretation: LH, YQH, XC, XMH, HHW, YZ, and CHL. Manuscript writing: all authors. All authors contributed to the article and approved the submitted version.

## Funding

This work was supported by the National Key Research and Development Plan of China (No. 2021YFF1201003), the Key R&D Program of Guangdong Province, China (No. 2021B0101420006), the National Science Fund for Distinguished Young Scholars (No. 81925023), the National Natural Scientific Foundation of China (No. 81901910, 81771912, 82071892, 82072090, and 82001986), the High-level Hospital Construction Project (DFJHBF202105).

## Conflict of Interest

The authors declare that the research was conducted in the absence of any commercial or financial relationships that could be construed as a potential conflict of interest.

## Publisher’s Note

All claims expressed in this article are solely those of the authors and do not necessarily represent those of their affiliated organizations, or those of the publisher, the editors and the reviewers. Any product that may be evaluated in this article, or claim that may be made by its manufacturer, is not guaranteed or endorsed by the publisher.

## Abbreviations

IHC, immunohistochemistry; NSCLC, non-small cell lung cancer; IMPRS, immune-based prognostic risk score; OS, overall survival; NCCN, National Comprehensive Cancer Network; AJCC/UICC, American Joint Committee Cancer/Union for International Cancer Control; TIL, tumor-infiltrating lymphocytes; FFPE, formalin-fixed paraffin embedded; IM, invasive margin; TC, tumor center; ROC, receiver operating characteristic; C-index, concordance index; iAUC, integrated area under the ROC curve; iBS, integrated Brier score; NRI, net reclassification improvement; IDI, integrated discrimination improvement; IQR, interquartile range.
