# Supplementary material for: Development and Validation of an Immune-Based Prognostic Risk Score for Patients With Resected Non-Small Cell Lung Cancer
Source: Front Immunol. 2022 Mar 23;13:835630. doi: 10.3389/fimmu.2022.835630 (PMC8983932; doi:10.3389/fimmu.2022.835630)
Supplement: Supplementary file 1 [file DataSheet_1.docx]

**Title:** Development and validation of an immune-based prognostic risk score for patients with resected non-small cell lung cancer

**Appendix:**

**Appendix A1. Immunohistochemical (IHC) staining for tissue sample**

Initially, we selected one tumor block containing both the tumor center (TC) and invasive margin (IM) of NSCLC from each patient for immunohistochemical (IHC) staining in both discovery and external validation cohorts. Three representative tissue paraffin section of 4𝛍𝗺 was processed for IHC staining of CD3^+^, CD4^+^, and CD8^+^ T cells. Then, tumor cross section slides were stained using a Ventana Discovery XT automated system (Ventana Medical System, Tucson, AZ) as per manufacturer’s protocol with recommended reagents. Briefly, slides were deparaffinized with EZ Prep solution (Ventana) and a heat-induced antigen retrieval method was used mild cell conditioning using CC1 antigen retrieval buffer (Ventana). A rabbit primary antibody for CD3, CD4, and CD8 (790-4341, Ventana) was used to supplied concentration and incubated for 16 min. Next, a Ventana OmniMap Anti-Rabbit Secondary Anbibody was applied to the samples for 16 min, and the Ventana ChromoMap kit was used as the detection system. Slides were then counterstained with Hematoxylin and dehydrated. Finally, the slides were cover slipped as per normal laboratory protocol.

After the IHC staining, one board-certified lung cancer pathologist (Y.LX) selected three representative regions of interest (ROI) of 230 µm × 230 µm for each slides at 200X magnification in both TC and IM for the following analysis, respectively.

**Appendix A2. The algorithms of IHC-immune features extraction**

In total, 5580 quantitative features were extracted for each patient, which included intensity and texture features. The features of intensity and texture features were extracted without/after a filtration of the Laplacian of Gaussian filter (filter parameter = 1.0, 1.5, 2.0, 2.5, respectively) from each RGB channel of the IHC-staining image.

1. **Laplacian of Gaussian filtration for the intensity and texture features**

The Laplacian of the Gaussian filter ($\nabla^{2}G$) distribution is given by

$$\nabla^{2}G\left( x,y \right)=\frac{-1}{\pi\sigma^{4}}(1-\frac{x^{2}+y^{2}}{2\sigma^{2}})e^{-(\frac{x^{2}+y^{2}}{2\sigma^{2}})}$$

$x,y$ denote the spatial coordinates of the pixel, and $\sigma$ is the value of the filter parameter.

1. **Intensity features:**

$X(i)$ indicates the intensity of the gray level $i$, and $N$ denotes the sum of the pixels in the image.

*1) Kurtosis*

$$kurtosis=\frac{\frac{1}{N}\sum_{i=1}^{N} {(X\left( i \right)-\bar{X})}^{4}}{{(\sqrt{\frac{1}{N}\sum_{i=1}^{N} {(X\left( i \right)-\bar{X})}^{2}})}^{4}}$$

*2) Skewness*

$$skewness=\frac{\frac{1}{N}\sum_{i=1}^{N} {(X\left( i \right)-\bar{X})}^{3}}{{(\sqrt{\frac{1}{N}\sum_{i=1}^{N} {(X\left( i \right)-\bar{X})}^{2}})}^{3}}$$

1. **Texture features:**

A matrix $P\left( i,j \right)$ to indicate the relative frequency with the intensity values of two pixels ($i$ and $j$) at the three distances (δ=1,2,3) and in four directions (0º，45º，90º，135º). $N_{g}$is the number of discrete intensity levels in the image;$x,y$ denote the spatial coordinates of the pixel. $\mu$, $\mu_{x}\left( i \right), \mu_{y}(j)$ is the mean of $P(i,j)$,$P_{x}(i)$,$P_{y}(j)$, and $\sigma_{x}(i)$, $\sigma_{y}(j)$is the standard deviation of $P_{x}(i)$, $P_{y}(j)$, respectively.

*1) Contrast*

$$contrast=\sum_{i=1}^{N_{g}} \sum_{j=1}^{N_{g}} \left| i-j \right|^{2}P(i,j)$$

*2) Correlation*

$$correlation=\frac{\sum_{i=1}^{N_{g}} \sum_{j=1}^{N_{g}} ijP\left( i,j \right)-\mu_{i}\left( i \right)\mu_{j}\left( j \right)}{\sigma_{x}\left( i \right)\sigma_{y}\left( j \right)}$$

*3) Entropy*

$$entropy=-\sum_{i=1}^{N_{g}} \sum_{j=1}^{N_{g}} P\left( i,j \right)\log\left[ P\left( i,j \right) \right]$$

*4) Energy*

$$energy=\sum_{i=1}^{N_{g}} \sum_{j=1}^{N_{g}} \left[ P\left( i,j \right) \right]^{2}$$

*5) Homogeneity*

$$homogeneity=\sum_{i=1}^{N_{g}} \sum_{j=1}^{N_{g}} \frac{P\left( i,j \right)}{1+\left| i-j \right|^{2}}$$

**Appendix A3. R packages used in this study**

The LASSO Cox regression model was done using the “glmnet” package, and the time-dependent receiver operating characteristic (ROC) analyses were done using the “survivalROC” package. The multivariable Cox regression analysis, calibration plots, and the nomogram plots were done with the “rms” package. The calculation of the C-index was performed with the “Hmisc” package, and the calculation of net reclassification index (NRI) and integrated discrimination improvement (IDI) were performed with the “survIDINRI” package. The calculation of the integrated area under the ROC curve (iAUC), the time-dependent AUC (tAUC), and the integrated Brier score (iBS) were performed with the “risksetROC” package. The internal validation of the C-index was performed with the “rms” package. The acquisition of the decision curve analysis was performed with the “stdca.R” package.

**Appendix A4. The calculation of the IHC-immune signature for each patient**

$$Score of IHC-immune signature=0.092\times CD3\_TC\_B\_entropy\_0\_3\_1.5+0.000001\times CD3\_TC\_B\_entropy\_45\_3\_1.5-0.160\times CD3\_TC\_G\_correlation\_45\_2\_0+0.020\times CD3\_TC\_R\_kurtosis\_0-0.142\times CD3\_TC\_R\_correlation\_45\_1\_0+0.145\times CD8\_IM\_R\_kurtosis\_0+0.059\times CD8\_IM\_R\_kurtosis\_1.0-0.034\times CD8\_TC\_G\_energy\_90\_1\_0$$

**Appendix Figures:**

**Appendix Figure A1.** LASSO Cox regression analysis for IHC-immune features selection.

**Appendix Figure A2.** Kaplan-Meier survival curves for patients with stage I NSCLC. Kaplan-Meier survival curves for patients with stage I NSCLC in the discovery (A) and validation cohort (B) according to the immunohistochemistry (IHC)-immune signature.

**Appendix Figure A3.** Kaplan-Meier survival curves for patients that received adjuvant chemotherapy and those that did not receive adjuvant chemotherapy. Kaplan-Meier survival curves for patients that received adjuvant chemotherapy in the discovery (A) and validation cohort (B) according to the immunohistochemistry (IHC)-immune signature; and Kaplan-Meier survival curves for patients that did not receive adjuvant chemotherapy in the discovery (C) and validation cohort (D) according to the immunohistochemistry (IHC)-immune signature.

**Appendix Tables:**

**Appendix Table A1.** IHC-based image features extracted from each patient

| **Type** | **Detail** |  |
| --- | --- | --- |
| **Intensity** | $\alpha$_$\gamma$_$\theta$_kurtosis_$\sigma$ | $\alpha$_$\gamma$_$\theta$_skewness_$\sigma$ |
| **Texture feature** | $\alpha$_$\gamma$_$\theta$_contrast_$\beta$ _ δ _$\sigma$ | $\alpha$_$\gamma$_$\theta$_correlation_$\beta$ _ δ _$\sigma$ |
|  | $\alpha$_$\gamma$_$\theta$_energy_$\beta$ _ δ _$\sigma$ | $\alpha$_$\gamma$_$\theta$_homogeneity_$\beta$ _ δ _$\sigma$ |
|  | $\alpha$_$\gamma$_$\theta$_entropy_$\beta$ _ δ _$\sigma$ |  |

**Note.** $\alpha$ represents the types of T cells, which could be CD3, CD4, and CD8. $\gamma$ represents tumor regions, which could be TC and IM. $\theta$ represents RGB color channels, which could be R, G, and B. $\beta$ represents the directions, which could be 0º, 45º, 90º, and 135º. $\sigma$ represents the filter value applied, which could be 1.0, 1.5, 2.0, and 2.5. When $\sigma$=0, features were extracted without filtration; δ represents the considered distances, which could be 1, 2, and 3. IHC, immunohistochemistry; TC, tumour centre; IM, invasive margin; R, red; G, green; B, blue.
